# Bovine Leptospirosis Due to Persistent Renal Carriage of *Leptospira borgpetersenii* Serovar Tarassovi

**DOI:** 10.3389/fvets.2022.848664

**Published:** 2022-04-05

**Authors:** Camila Hamond, Karen LeCount, Ellie J. Putz, Darrell O. Bayles, Patrick Camp, Marga G. A. Goris, Hans van der Linden, Nathan E. Stone, Linda K. Schlater, Jason W. Sahl, David M. Wagner, Jarlath E. Nally

**Affiliations:** ^1^National Veterinary Services Laboratories, Animal and Plant Health Inspection Service (APHIS), United States Department of Agriculture, Ames, IA, United States; ^2^NCAH Leptospira Working Group, United States Department of Agriculture, Ames, IA, United States; ^3^Infectious Bacterial Diseases Research Unit, Agricultural Research Service (ARS), United States Department of Agriculture, Ames, IA, United States; ^4^Department of Medical Microbiology and Infection Prevention, Office International des Epizooties (OIE) and National Collaborating Centre for Reference and Research on Leptospirosis, Amsterdam University Medical Center, University of Amsterdam, Amsterdam, Netherlands; ^5^Department of Biological Sciences, The Pathogen and Microbiome Institute, Northern Arizona University, Flagstaff, AZ, United States

**Keywords:** *Leptospira*, *L. borgpetersenii*, leptospirosis, bovine, Tarassovi

## Abstract

Leptospirosis is a global zoonotic disease that causes significant morbidity and mortality in human and animal populations. *Leptospira interrogans* is a leading cause of human disease, and *L*. *borgpetersenii* is a leading cause of animal disease. Cattle are reservoir hosts of *L*. *borgpetersenii* serovar Hardjo, which is transmitted *via* urine, semen, and uterine discharges resulting in abortion and poor reproductive performance. Bovine bacterin vaccines can only protect against those serovars included in vaccine formulations and typically include serovar Hardjo among others. Genotyping and serotyping represent two different and unique methods for classifying leptospires that do not always correlate well; comprehensive characterization using either method requires recovery of isolates from infected animals. In this study, we report for the first time, isolation of *L*. *borgpetersenii* serovar Tarassovi from the urine of a dairy cow in the U.S. The classification of the isolate, designated strain MN900, was confirmed by whole-genome sequencing, serotyping with reference antisera and monoclonal antibodies, Matrix Assisted Laser Desorption/Ionization (MALDI), and immunoblotting with reference antisera. Strain MN900 was excreted in urine samples for 18 weeks even as the cow was seronegative for serovar Tarassovi. Strain MN900 has an unusual morphology since it is not as motile as other leptospires and lacks hooked ends. Serovar Tarassovi is not included in U.S. bacterin vaccines. These results demonstrate the importance of culture and concomitant genotyping and serotyping to accurately classify leptospires, and as required to design efficacious vaccine and diagnostic strategies to not only limit animal disease but reduce zoonotic risk.

## Introduction

Leptospirosis is a neglected zoonotic disease of worldwide importance caused by pathogenic spirochetes belonging to the genus *Leptospira*; it causes significant morbidity and mortality in both human and domestic animal populations (1, 2). Bovine leptospirosis can significantly impact production on infected farms due to infertility, abortions, stillbirths, weak offspring, and decreased milk production and growth rates (3, 4). Leptospires can be isolated from kidneys and the reproductive tract of infected cattle and are shed directly to other cattle in urine, semen, or uterine discharges. Transmission of disease to humans typically occurs *via* direct contact with infected urine or indirectly through the contaminated environment (4). Livestock farming and abattoir workers have occupational risk factors due to exposure to pathogenic *Leptospira* species from cattle (3, 5, 6).

Seroprevalence studies conducted in the U.S. more than 20 years ago indicated that cattle were exposed to multiple serovars of *Leptospira*, including Hardjo, Pomona, Icterohaemorrhagiae, Canicola, Grippotyphosa, Pyrogenes, Ballum, Autumnalis, Tarassovi, and Australis (7). More recent studies indicate that U.S. cattle are also exposed to serovar Bratislava, though whether it causes bovine leptospirosis remains to be determined (8). Bovine leptospirosis in the U.S. is typically associated with *L*. *borgpetersenii* and *L*. *interrogans*, and a range of serovars including Hardjo, Pomona, and Grippotyphosa which have been isolated from kidneys and urine of infected animals (7–9). Globally, cattle are recognized as a reservoir host for *L. borgpetersenii* serovar Hardjo (4). In South America, bovine leptospirosis is also caused by *L. santarosai* (10, 11) and *L. noguchii* (11–13).

Genotyping and serotyping represent two different and unique methods for classifying leptospires that do not correlate well with each other (14). Serotyping is dependent on surface antigens of *Leptospira* including lipopolysaccharide (LPS), a protective antigen that mediates protection in bacterin vaccines. Since these surface antigens can vary among infecting strains (resulting in hundreds of different serovars), accurate and complete serotyping of isolates recovered from livestock animals is fundamental to the development and use of efficacious bacterins, surveillance, and epidemiology of this insidious disease (4). Thirty-eight species of pathogenic *Leptospira* (clade P1 and clade P2) have been described to date (15).

Bacterin vaccines are designed to reduce bovine leptospirosis by including those serovars associated with bovine disease and typically include serovars such as Hardjo, Pomona, and Grippotyphosa. But to ensure current bovine vaccines are efficacious, it is essential to survey infected animals and characterize recovered isolates to identify all species and serovars involved in bovine disease. In this study, a U.S. dairy cow was found to be shedding *L*. *borgpetersenii* serovar Tarassovi, a serovar that has neither previously been cultured in the U.S. nor isolated from the urine of a dairy cow.

## Materials and Methods

### Dairy Cow #MN900

Several cows in a dairy farm in Minnesota, U.S., with a history of poor reproductive performance (failure to breed and maintain early pregnancy) were diagnosed as actively shedding pathogenic leptospires using *lipL32* qPCR. One of these PCR-positive cows, a 3.5-year-old Holstein designated MN900, was purchased to facilitate follow-up research studies including the culture of urine and detection of persistent carriage of leptospires over time. A detailed medical history for MN900 prior to purchase was not available. MN900 was considered healthy, and no clinical signs of disease were observed during a 2-week quarantine period with daily evaluation by clinical veterinarians. MN900 was housed in a field barn with access to pasture, grass hay, and water *ad libitum*, which was supplemented with a grain/concentrate mix. All animal experimentations were conducted in accordance with protocols as reviewed and approved by the Animal Care and Use Committee at the National Animal Disease Center, and as approved by USDA Institutional guidelines.

### Sample Collection

Blood samples for serology were collected by jugular venipuncture into evacuated tubes (Vacutainer® BD Diagnostics, Franklin Lakes, NJ, USA). In addition to the original urine sample collected before arrival at the National Animal Disease Center, the first and second urine voids were collected from dairy cow MN900 at weekly intervals for 23 weeks for a total of 47 urine samples. Before urine collection, the vulva was washed with sterile water to remove gross debris. Hair-clippers were used to trim excess hair before an additional wash with sterile water and a final rinse with 70% ethanol. A diuretic was administrated by intravenous injection and midstream urine from a first and second void was collected into sterile containers.

### Microscopic Agglutination Test

The microscopic agglutination test (MAT) was performed according to World Organization for Animal Health guidelines using a panel of 19 antigens representative of 15 serogroups (Supplementary Table S1) (16). A titer was considered positive at ≥1:100.

### Culture

A 1-ml aliquot of freshly collected urine was immediately inoculated into 9 ml of transport Hornsby and Nally (HAN) media. Within 1 h, 200 μl of urine, as well as 200 μl of inoculated transport HAN media, was used to inoculate 5 ml HAN liquid, 5 ml HAN semi-solid, and 5 ml T80/40/LH semi-solid media. Inoculated T80/40/LH media was incubated at 29°C and inoculated HAN media was incubated at both 29 and 37°C in 3% CO_2_ (17, 18). Inoculated tubes were examined daily by darkfield microscopy.

### Fluorescent Antibody Testing and qPCR

A 40 ml aliquot of urine was centrifuged at 10,000 × *g* for 30 min at 4°C. The supernatant was removed, and the urinary pellet was resuspended in 2 ml PBS. The resuspended sample was split in two and washed by centrifugation at 12,000 × *g* for 10 min at 4°C. The supernatant was removed until ~150 μl remained and this was resuspended in 500 μl PBS. The pellet was again harvested by centrifugation at 12,000 × *g* for 10 min at 4°C. The supernatant was removed until approximately 100 μl remained; one sample was used for FAT and one sample was used for qPCR.

Fluorescent antibody testing was performed in duplicate as previously described (8). For qPCR, DNA was extracted from the urinary pellet using the Maxwell RSC Purefood Purification Pathogen kit (Promega Corporation, Madison, WI, U.S.A), following the manufacturer's instructions with 1 h of incubation with lysis buffer A and a 100 μl elution volume. DNA from *L. borgpetersenii* serovar Hardjo strain HB203 was used for a standard curve. The DNA was quantified using Qubit (Qubit dsDNA BR assay, Qubit 3.0 fluorometer, Invitrogen, Waltham, MA, U.S.A). Genome size of 3.67 Mb was used to determine the genomic equivalent (GEq) concentration per microliter of the purified DNA. To generate a standard curve, serial dilutions of the DNA were made starting at 1 × 10^7^ GEq to 1 × 10^0^ GEq/5 μl (19). Standard-curve assays were performed in triplicate. The concentration of leptospires in urine samples was quantified using a TaqMan-based quantitative PCR assay with a QuantStudio™ 7 Flex Real-Time PCR System (Thermo Fisher, Waltham, MA, U.S.A). The *lipL32* gene was amplified using a set of primers and protocol as described previously: LipL32-47Fd (5′-GCATTACMGCTTGTGGTG-3′) and LipL32-301Rd (5′-CCGATTTCGCCWGTTGG-3′), the probe LipL32-189P (6-carboxyfluorescein [FAM]-5′-AA AGC CAG GAC AAG CGC CG-3′-black hole quencher 1 [BHQ1]), using PerfeCTa qPCR ToughMix®, Low ROX™ (Quanta Biosciences, Gaithersburg, MD, USA) (20, 21). As a control for PCR inhibitors, the TaqMan® Exogenous Internal Positive Control was added to the master mix to confirm DNA amplification and detect false negatives and to qualitatively detect the presence of amplification inhibitory substances in a sample. All samples were assayed in triplicate. The sample was considered positive when duplicate or triplicates were positive with Ct values <40. The bacterial quantification in urine samples was calculated as previously described (19).

### Genotyping of *Leptospira* Directly From Urine Samples

To determine the species of *Leptospira* excreted in urine samples from dairy cow MN900 that were positive by *lipL32* qPCR, the *secY* housekeeping gene was amplified with the primers *secY*_F (5′-ATGCCGATCATTTTTGCTTC-3′) and *secY*_R (5′-CCGTCCCTTAATTTTAGACTTCTTC-3′) (22) and nested primers SecYIVF (5′-GCGATTCAGTTTAATCCTGC-3′) and SecYIVR (5′-GAGTTAGAGCTCAAATCTAAG-3′) (23). After amplification, the PCR products were purified and labeled using the Big Dye Terminator v3.1 cycle sequencing reagent (Applied Biosystems, Waltham, MA, USA). Sequencing was performed using the ABI 3130XL Genetic Analyzer. Sequence data were analyzed with DNAStar Lasergene sequence analysis software. Consensus sequences were then compared with available sequences in the GenBank database using BLAST. A phylogenetic tree was made with Geneious Prime 2020.2.2 using the neighbor-joining method, with the Tamura-Nei nucleotide substitution model (https://www.geneious.com/).

### Typing of *Leptospira*

#### Genome Sequencing

For Illumina Genome Sequencing, DNA was extracted from a 5 ml culture of strain MN900 using the Maxwell RSC Purefood Purification Pathogen kit (Promega Corporation, Madison, WI), following instruction from the manufacturer. For Nanopore Genome Sequencing, DNA was extracted from a 5 ml culture of strain MN900 using the Nanobind CBB Big DNA Kit–Beta Handbook v1.8 (07/2019) (Circulomics, Baltimore, MD, U.S.A.). The concentration of the reconstituted genomic DNA was determined using the Qubit dsDNA Broad Range Assay Kit to ensure that there was a minimum of 25 ng/μl for Nanopore and 1 ng/μl for Illumina sequencing, and purity was assessed using the NanoPhotometer Pearl® (IMPLEN, Westlake Village, CA, U.S.A.).

Illumina (MiSeq Desktop Sequencer, 2 × 250 v2 paired-end chemistry, and the Nextera XT DNA Library Preparation Kit, Illumina, San Diego, CA, U.S.A.) genome sequence was obtained as per instruction from the manufacture. Before Nanopore sequencing, purified DNA was passed through the Circulomics Short Read Eliminator Kit XS following instruction from the manufacturer. DNA was again quantified using the Qubit dsDNA Broad Range Assay Kit and 1 μg was used. The Native barcoding genomic DNA Kit was used following instruction from the manufacturer. Samples were pooled in equal amounts and loaded onto a Nanopore flowcell FLMIN106. The flowcell was run for 12 h.

Before assembly, the Oxford Nanopore reads were subset to reads ≥ 40 Kb, which resulted in a total of 69 × long-read genome coverage. Additionally, 164 × coverage of Illumina paired-end reads was also used for error correction in the post-assembly processing. Genome assembly and error correction was accomplished with Unicycler v0.4.7 (24) and the default settings except that a *Leptospira* ParA protein sequence was added to the start genes database to ensure that the small chromosome would be rotated to start at the *parA* gene. The genome annotation was completed by the NCBI Prokaryotic Genome Annotation Pipeline (25).

To place the strain MN900 within the larger phylogenetic framework of *L. borgpetersenii*, all publicly available genome assemblies for this species were downloaded from GenBank on 29 October 2021 using the NCBI genome-download tool (https://github.com/kblin/ncbi-genome-download). All genomes were aligned against *L. borpetersenii* serovar Ballum strain 56604 (GCA_001444465.1) with nucmer v4.0.0 (26) and Single nucleotide polymorphism (SNPs) were called with NASP v1.20 (27). SNPs that fell within duplicated regions, based on a reference self-alignment with nucmer, were filtered from downstream analyses. SNPs that fell within five positions of each other were filtered from the analysis, which has been demonstrated to decrease the number of false positive SNP calls (28). The remaining SNPs were queried from *L. mayottensis* strain 200901116 (GCA_000306675.3) for rooting. A maximum-likelihood phylogeny was inferred on the concatenated SNP alignment with IQ-TREE v2.0.3 (29) using the integrated ModelFinder method (30) and 1,000 bootstraps.

#### Speciation by MALDI-TOF

Strain MN900 was cultured in HAN media and samples prepared for the MALDI Autoflex® Speed LRF (Bruker, Billerica, MA, USA) to identify species, as previously described (31). An identification score ≥2.3 was considered valid for identification at the species level (32).

#### Serotyping

Strain MN900 was serotyped using the MAT method with a panel of polyclonal rabbit reference antisera representing thirteen serogroups: Australis, Autumnalis, Ballum, Bataviae, Canicola, Grippotyphosa, Hebdomadis, Icterohaemorrhagiae, Mini, Pomona, Pyrogenes, Sejroe, and Tarassovi (Supplementary Table S2). The isolate was further typed at the serovar level by performing MAT with panels of monoclonal antibodies (mAbs) that characteristically agglutinate serovars from the serogroup Tarassovi (F151C1, F151C6, F151C7, F151C8, F151C9, F151C13, F151C17, F151C19, and F151C20) as previously described (33, 34).

### Morphology

#### Dark-Field Microscopy

*L. borgpetersenii* serovar Tarassovi strain MN900 (this study), serovar Hardjo strain TC129 (8), and serogroup Ballum strain LR131 (Hamond et al., unpublished data) were cultured in HAN media at 29°C to mid-late (1–3 × 10^8^ leptospires/ml) log phase. A 10 μl aliquot of cells was placed onto glass slides, coverslipped, and visualized using dark-field microscopy. Images were recorded with an Infinity five digital camera (Lumenera, Ottawa, Ontario, Canada) and Leica Application Suite v 3.4.0 software (Leica Microsystems, Wetzlar, Germany), while the motility was recorded with a Leica CH-9435 Heerbrugg and Leica Application Suite v 3.4.0 software.

#### Scanning Electron Microscopy

About 1 ml containing 1 × 10^6^ strain MN900 in HAN media was used to inoculate each well of a 24-well plate (flat bottom, low evaporation lid, Corning Inc., Corning, NY, U.S.A.) containing a sterile 12 mm glass coverslip (12 × 12 mm). Plates were incubated at 37°C in 3% CO_2_ and after 24 h, the supernatant was removed, and the coverslip fixed in 4% PFA/2.5% glutaraldehyde in 0.1 M cacodylate buffer for 15 min. The samples were stained by sequential exposure to osmium and thiocarbohydrazide. Samples were dehydrated through graded alcohols and chemically dried with hexamethyldisilizane. Samples were coated with a gold/palladium mixture and viewed on the Hitachi TM3030Plus SEM (Hitachi, U.S.A).

### Gel Electrophoresis and Immunoblots

Leptospires (mid-late log phase, 1–3 × 10^8^ leptospires/ml) were harvested by centrifugation (10,000 × *g*, 4°C, 30 min), washed twice with Phosphate buffered saline (PBS), and processed for one-dimensional (1-D) Sodium dodecyl-sulfate polyacrylamide gel electrophoresis (SDS-PAGE) on 12% acrylamide gels (BioRad, Hercules, CA, U.S.A.) as per the guidelines by the manufacturer. Proteins were visualized by staining with Sypro Ruby (Invitrogen) and LPS was visualized by staining with Pro-Q Emerald 300 (Invitrogen) as per manufacturer's guidelines. For immunoblotting, samples were transferred by semi-dry transfer (Amersham TE77 PWR) to Immobilon-P transfer membrane (Millipore, 220 Bedford, MA, U.S.A.) and blocked overnight at 4°C with Starting Block (PBS) blocking buffer (Thermo Fisher). Membranes were individually incubated with indicated antisera diluted in blocking buffer (anti-LipL32 at 1:4,000, or anti-Tarassovi, anti-Hardjo, or anti-Ballum at 1:1,000) followed by incubation with horseradish-peroxidase anti-rabbit immunoglobulin G (Sigma, St. Louis, MO, U.S.A) conjugate diluted 1:4,000 in blocking buffer. Bound conjugates were detected using Clarity Western ECL substrate (BioRad) and images were acquired using a Bio-Rad ChemiDoc MP imaging system.

## Results

### Detection of *Leptospira* in Bovine Urine

**Figure 1:**
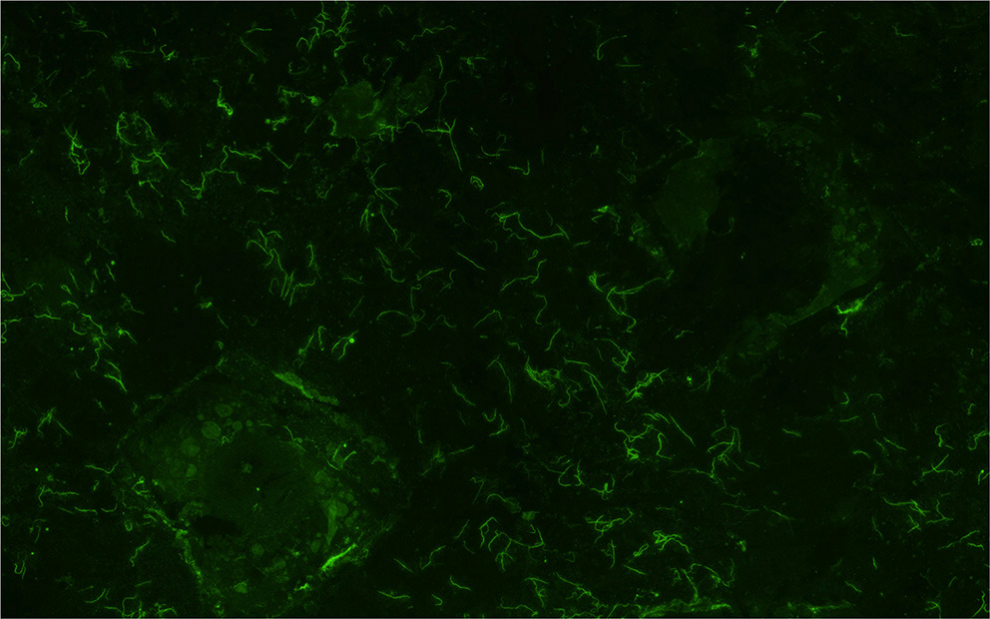
Representative image of a urine sample from dairy cow MN900 that was positive for leptospires (Sample collected week 2, void 2) by the fluorescent antibody test (FAT). All positive FAT samples are listed in Supplementary Table 3. Original magnification 400 ×.

**Figure 2:**
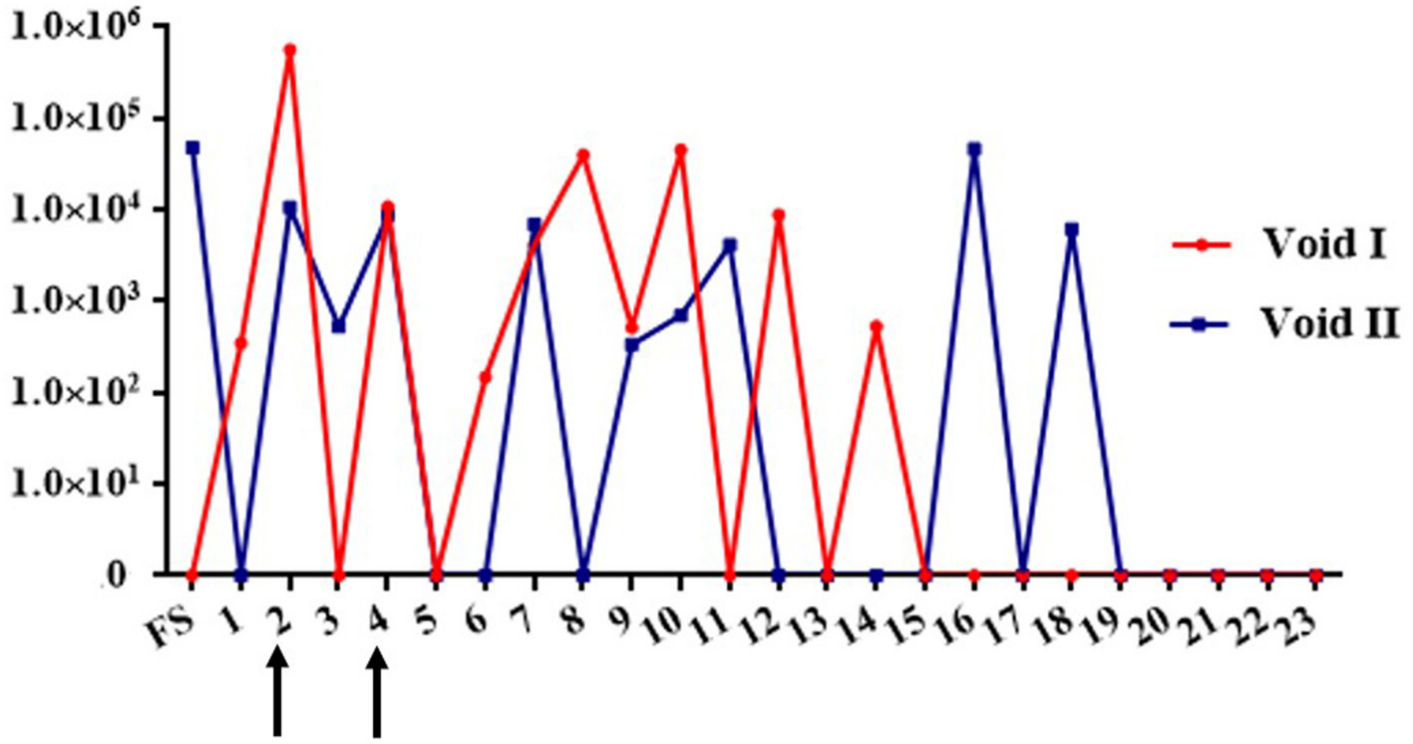
Graph depicting detection of urinary excretion of leptospires in dairy cow MN900 by qPCR in urine void 1 (red line) or void 2 (blue line). Arrows indicate time points at which urine was also positive by culture. Genome equivalent numbers are shown on the y-axis; week number of sample collection on site is shown on the x-axis. FS, Farm sample.

Eight (17%; 8/47) urine samples were positive by FAT, 5/23 (21.7%) in void 1 and 3/24 (12.5%) in void 2 (Table 1, Figure 1). PCR determined that twenty (42.5%; 20/47) urine samples were positive for the presence of *lipL32*, 10/23 (43.48%) in void 1, and 10/24 (41.67%) in void 2 (Table 1). Overall, detection of leptospires excreted in urine over the course of 6 months indicated that shedding was intermittent and was no longer detectable after week 18 (Figure 2).

**Table 1:** Detection of *Leptospira* in urine samples (void 1 and void 2) from dairy cow MN900 by fluorescent antibody test (FAT), *lipL32* qPCR and culture.

**Urine ID**	**FAT**	**qPCR**	**Culture**
Void 1	5/23 (21.73%)	10/23 (43.48%)	2/23 (8.69%)
Void 2	3/24 (12.5%)	10/24 (41.67%)	2/23 (8.69%)
**Total**	**8/47 (17%)**	**20/47 (42.55%)**	**4/46 (8.69%)**

*Urine ID: Indicates if a first void 1 or void 2 urine sample was collected, FAT: Fluorescent antibody test, qPCR: quantitative polymerase chain reaction, Culture: Whether a positive culture was obtained from the urine samples, and Total: Combined results of both urine void 1 + void 2*.

Four *Leptospira* isolates were obtained by culture in HAN media at 37°C in 3% CO_2_ from urine collected at week 2 and week 4, from both void 1 and void 2 (Figure 2, Table 1). One additional positive culture from void 1 at week 8 was not sustainable. The void 1 sample collected at week 2 was also culture positive when incubated at 29°C (Supplementary Table S1). No cultures were recovered using T80/40/LH.

Thirteen (27.6%) urine samples were positive only by qPCR and two (4.2%) only by FAT; three samples were positive by culture, FAT, and qPCR; two samples were positive by both qPCR and culture, and three samples were positive by both qPCR and FAT. All data are presented in Supplementary Table S3.

### Serology

A total of 23 weekly sera samples were collected for testing by MAT. Serology demonstrated that 14/23 (60.8%) of the samples contained anti-*Leptospira* antibodies: Two samples were positive for Australis with a titer of 1:100 and twelve samples were positive to more than one serogroup with titers that ranged from 100 to 200 (Supplementary Table S3). Notably, no sera samples reacted with an isolate of strain MN900 that was cultured from the urine sample collected at week 2.

### Genotyping of *Leptospira* Directly From Urine Samples

**Figure 3:**
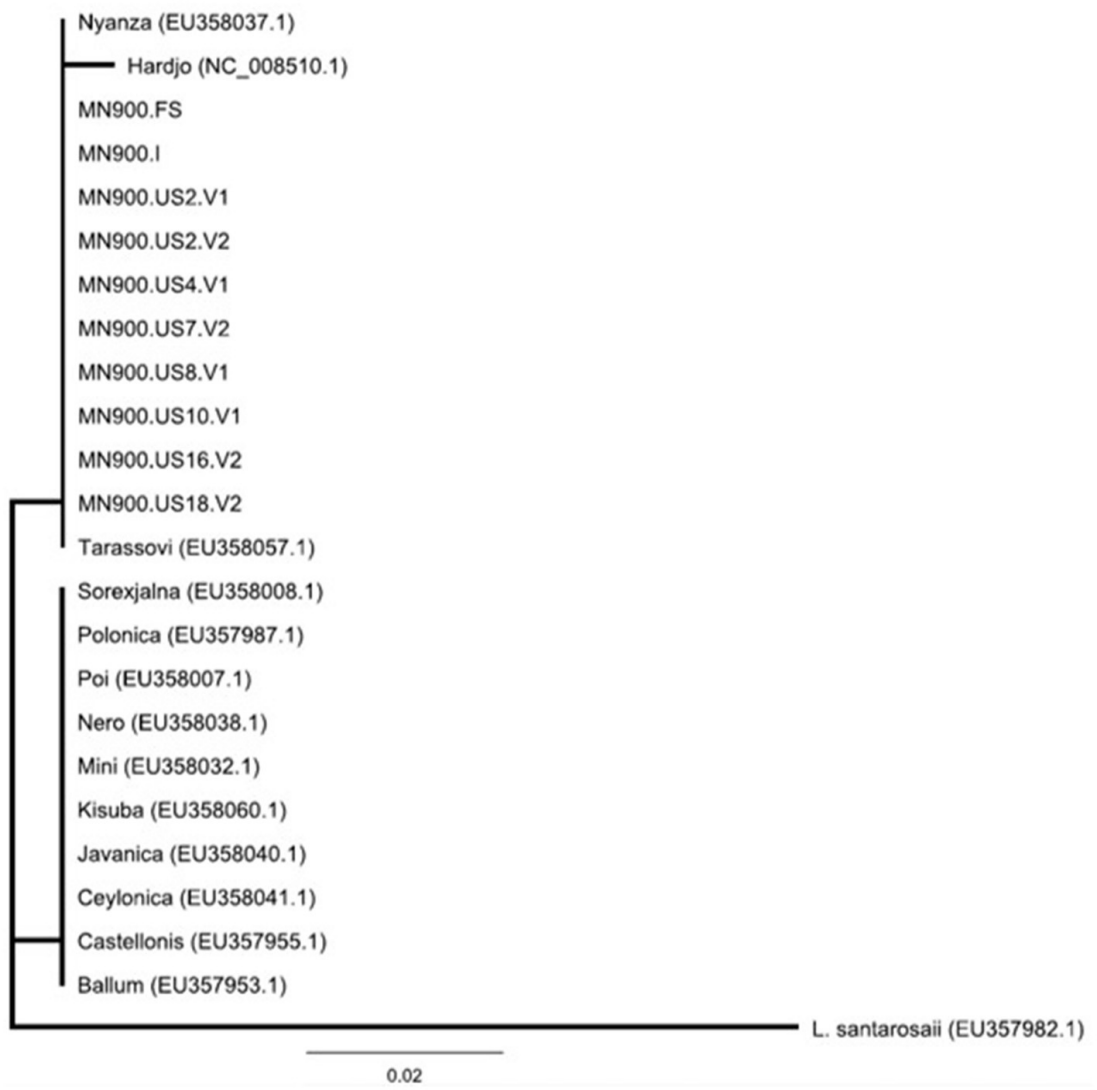
Phylogeny of *Leptospira* detected in the urine of cow MN900 and other *L. borgpetersenii* strains based on *secY* IV sequence analysis using the neighbor-joining method. *Leptospira* strain MN900 isolated from dairy cow MN900 is annotated as MN900.I and the urine sample collected prior to coming on site is annotated as MN900.FS (farm sample). Positive urine samples collected on site are annotated at MN900.US followed by the week number and whether it is urine void 1 (V1) or void 2 (V2). Accession numbers are provided for publicly available sequenced (NCBI) strains of *L. borgpetersenii* originally obtained from a variety of hosts.

Of the 20 urine samples positive by *lipL32* qPCR, 13 (65%) were positive by *secY* IV nested PCR. Of these, nine were of sufficient quality to obtain nucleotide sequence that was 100% identical to that of other *L. borgpetersenii* isolates when compared with GenBank/NCBI by BLAST. Phylogenetic analysis of *secY* IV sequences indicated that all MN900 clinical samples and the MN900 cultured isolate were identical to each other and representatives of *L. borgpetersenii* serovar Tarassovi (EU358057.1) and serovar Nyanza (EU358037.1) (Figure 3).

### Typing of *Leptospira* Strain MN900

#### MALDI-TOF Mass Spectrometry

Strain MN900 was typed as *L. borgpetersenii* with a score of 2.32.

#### Phylogenetic Analysis and WGS Details

**Figure 4:**
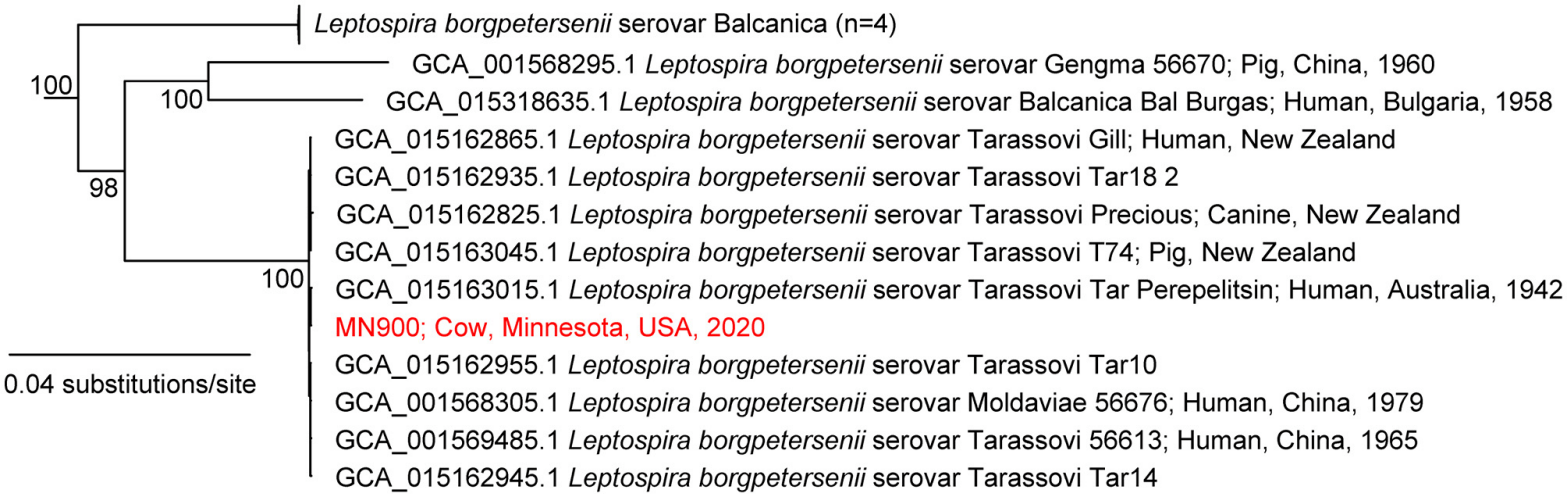
Maximum-likelihood phylogeny of *Leptospira borgpetersenii*, including strain MN900, based on complete whole genome sequence analysis. The phylogeny was inferred with IQ-TREE on a concatenation of 49,679 SNPs and the GTR+F+ASC+R2 nucleotide substitution model. The phylogeny was rooted with *L. mayottensis* 20090116. The complete phylogeny is provided in Supplementary Figure S1.

Phylogenetic analysis grouped the complete genome of strain MN900 together in a monophyletic clade with all eight *L*. *borgpetersenii* serovar Tarassovi isolates included in the phylogeny as well as the one that included *L*. *borgpetersenii* serovar Moldaviae (Figure 4, Supplementary Figure S1); serovars Tarassovi and Moldaviae are both in the Tarassovi serogroup. The clade containing these 10 strains is distinct in the WGS phylogeny—the branch leading to it contains 840 SNPs and has 100% bootstrap support; however, there is limited diversity within this clade (Figure 4). The final completed assembly of *L*. *borgpetersenii* strain MN900 is comprised of two circularized chromosomes, one is 3,708,359 bp and the other 355,051 bp, both with a G+C content of 40%. Annotated assemblies are available in GenBank, BioProject PRJNA769499, Accession numbers CP084914 and CP084915 for chromosome 1 and 2, respectively.

#### Serotyping

**Figure 5:**
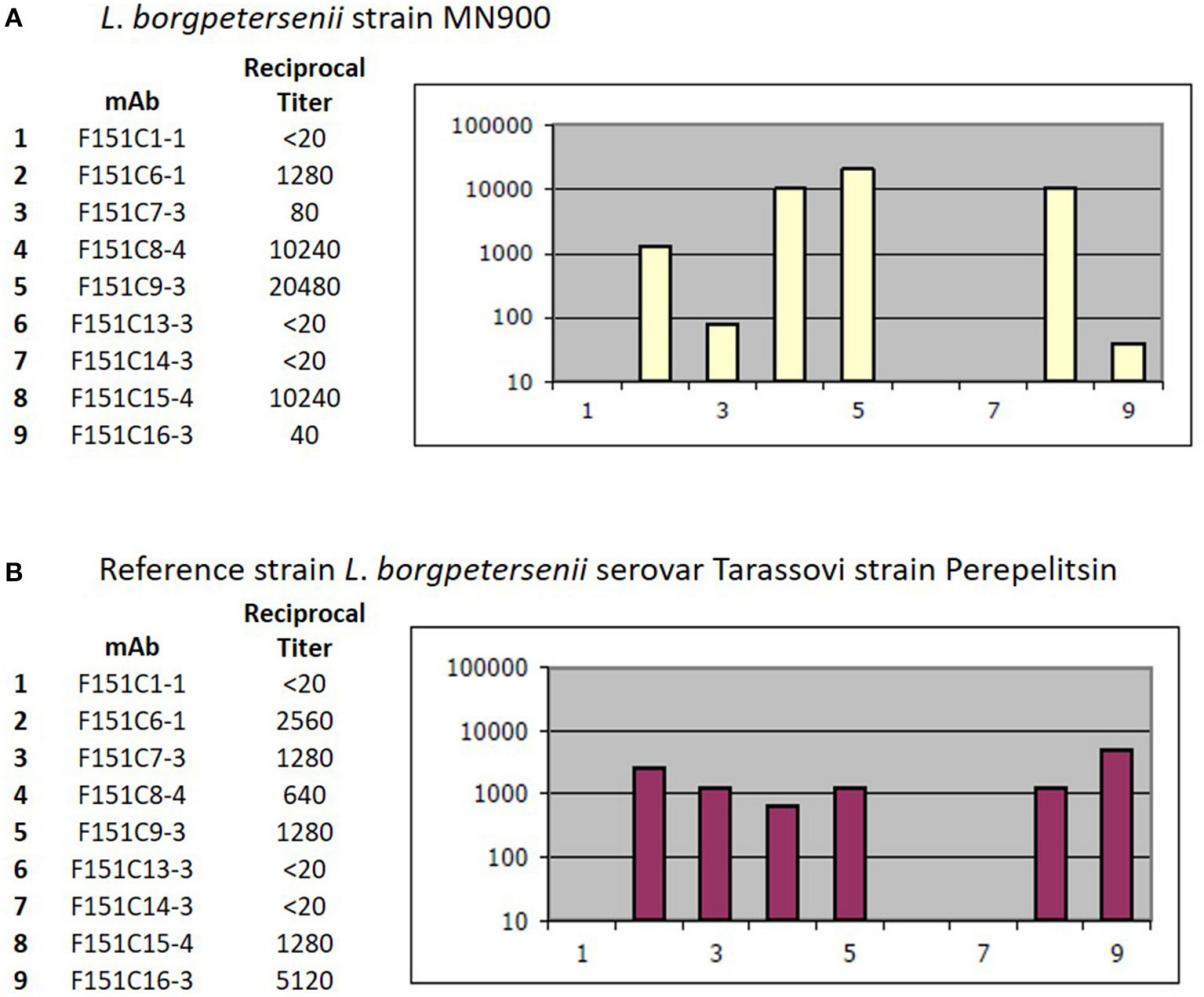
Serotyping with monoclonal antibodies (mAb) that characteristically agglutinate serovars from the serogroup Tarassovi (34). Titers of reactivity for each mAb are provided for **(A)** Strain MN900 and **(B)** the reference strain *L*. *borgpetersenii* serovar Tarassovi strain Perepelitsin. Reciprocal titers are shown on the y-axis; mAb number is shown on the x-axis.

Strain MN900 had high agglutination titers with reference antiserum specific for serogroup Tarassovi. Additional serotyping with monoclonal antibodies identified strain MN900 as belonging to serovar Tarassovi (Figure 5).

### Morphology and Motility

**Figure 6:**
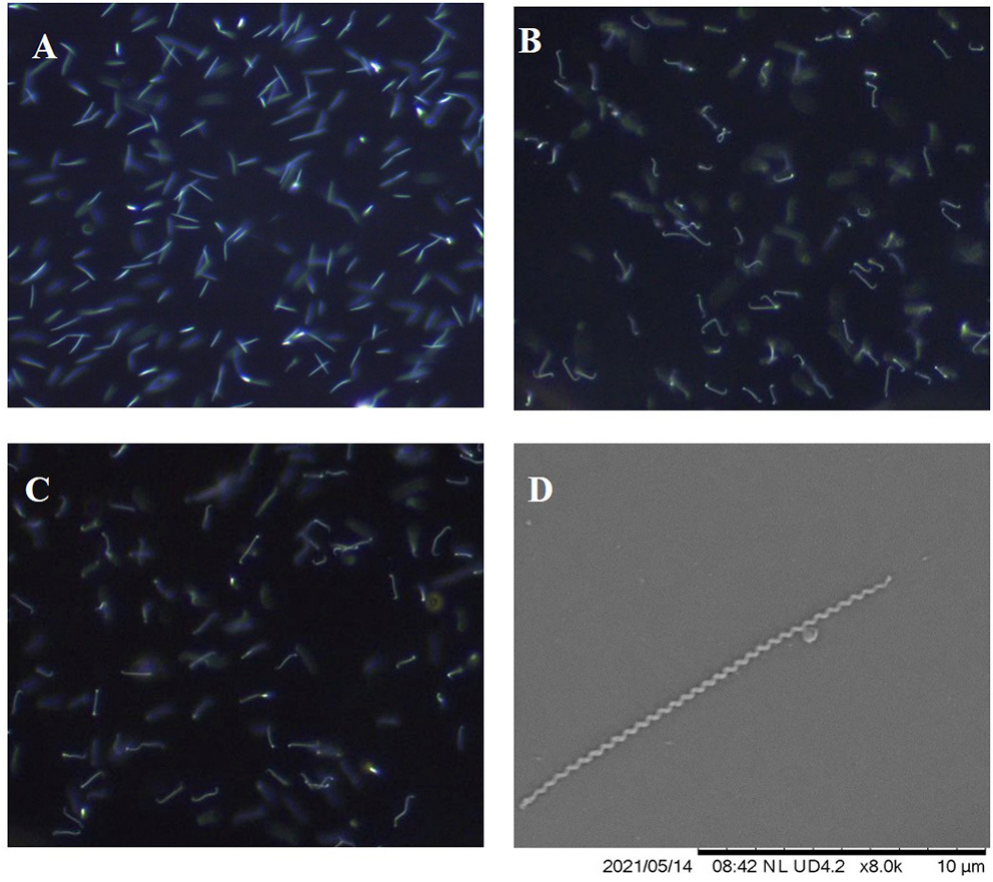
Representative images as visualized by dark-field microscopy of *L*. *borgpetersenii* **(A)** serovar Tarassovi strain MN900, **(B)** serovar Hardjo strain TC129 and **(C)** Serogroup Ballum strain LR131; original magnification ×200. **(D)** Scanning electron micrograph of *L*. *borgpetersenii* serovar Tarassovi strain MN900; original magnification ×8,000.

*L. borgpetersenii* serovar Tarassovi strain MN900 exhibited atypical morphology and motility for leptospires when viewed by dark-field microscopy (Figure 6A). When compared with *L*. *borgpetersenii* serovar Hardjo strain TC129 (Figure 6B) and *L*. *borgpetersenii* serogroup Ballum strain LR131 (Figure 6C), it does not show the classic hooked end, tight coiling, or motility. Representative video clips are provided demonstrating the limited motility of strain MN900 (Supplementary Video S1) compared to strain TC129 (Supplementary Video S2) or strain LR131 (Supplementary Video S3). Scanning electron microscopy confirmed the presence of a typical tightly coiled spiral-shaped morphology as observed in leptospires, but the absence of the classic hooked-end (Figure 6D).

### Proteins and Lipopolysaccharide

**Figure 7:**
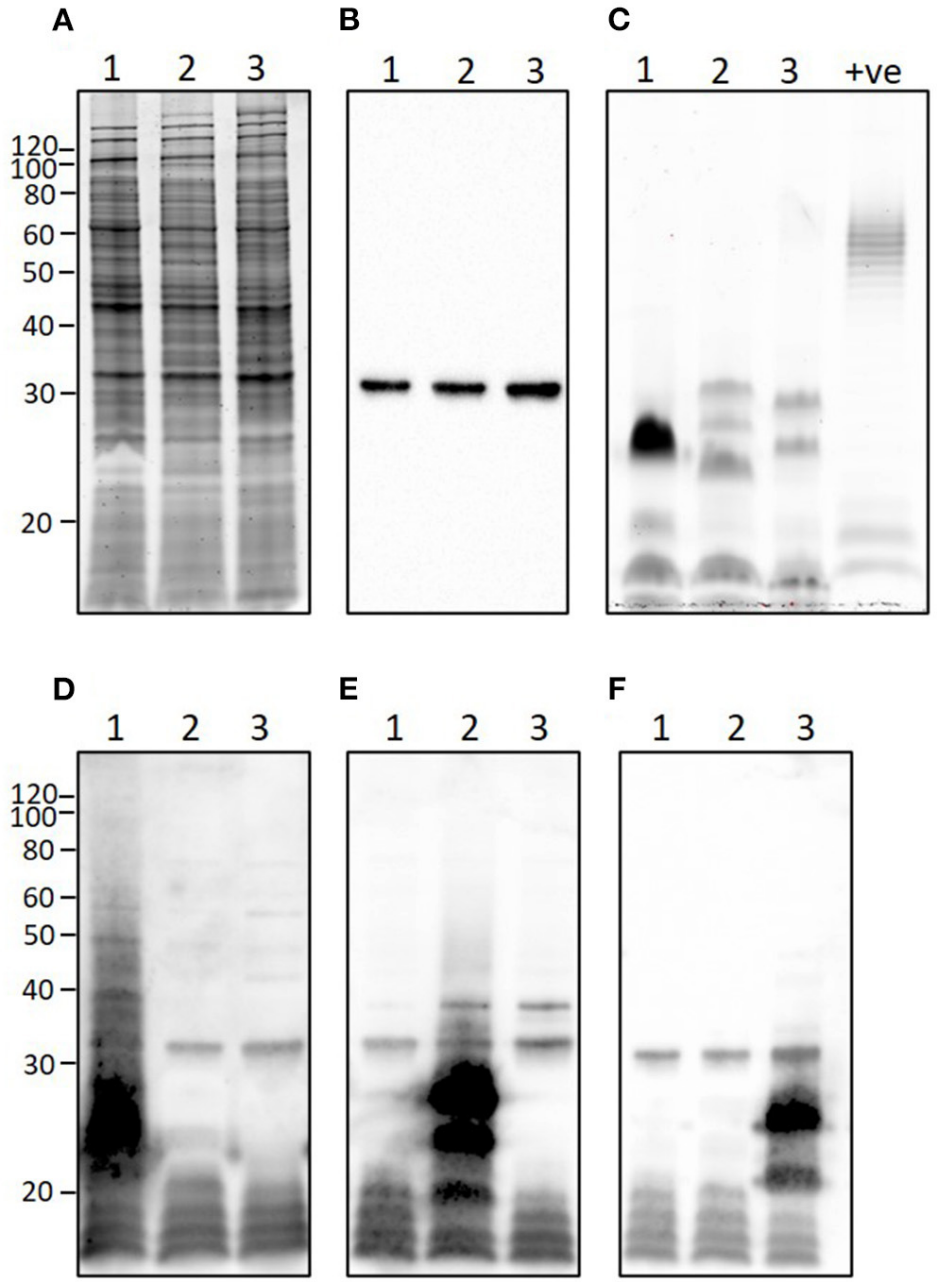
Representative images showing **(A)** total protein profile, **(B)** immunoblotting with anti-LipL32, **(C)** total lipopolysaccharide profile, and immunoblotting with **(D)** anti-Tarassovi, **(E)** anti-Hardjo or **(F)** anti-Ballum antisera of 1) *L*. *borgpetersenii* serovar Tarassovi strain MN900, 2) *L*. *borgeptersenii* serovar Hardjo strain TC129 and 3) *L*. *borgpetersenii* serogroup Ballum strain LR131. About 5 μg of each strain was loaded per lane. +ve; positive control for LPS staining comprising 5 μg of *E*. *coli* serotype 055:B5. Molecular mass markers are indicated.

*L*. *borgpetersenii* serovar Tarassovi strain MN900 has a similar protein profile to that of *L*. *borgpetersenii* serovar Hardjo strain TC129 and *L*. *borgpetersenii* serogroup Ballum strain LR131 (Figure 7A) and all three strains express the outer membrane lipoprotein LipL32 that is conserved among pathogenic *Leptospira* species (Figure 7B). In contrast, and as expected with strains of pathogenic leptospires belonging to different serovars and serogroups, each presented with a unique LPS profile (Figure 7C), as confirmed by immunoblotting with antisera specific for serovar Tarassovi, serovar Hardjo, and serovar Ballum (Figures 7D–F, respectively).

## Discussion

Viable leptospires were recovered from the urine of a 3.5-year-old U.S. dairy cow by culture in HAN media and were classified as *L. borgpetersenii* serovar Tarassovi using whole genome sequencing, serotyping with reference monoclonal antibodies, MALDI, and immunoblots with reference antisera. Although seroprevalence studies have demonstrated that U.S. cattle (7, 35) and deer (36) may be exposed to leptospires within the serogroup Tarassovi, this is the first report to demonstrate that *L. borgpetersenii* serovar Tarassovi was causing an active infection in a U.S. dairy cow, including persistent excretion *via* urine over several weeks.

Dairy cow MN900 was initially diagnosed as positive for leptospirosis based on a positive PCR specific for *lipL32*, a conserved gene unique to pathogenic leptospires. Additional PCR and sequencing of *secY* confirmed and diagnosed leptospirosis due to *L*. *borgpetersenii*, an unsurprising result given cattle are a known reservoir host for *L*. *borgpetersenii* globally, and that infection is associated with poor-reproductive performance (37, 38). However, since *secY* sequence cannot discriminate among or identify serovar status of leptospires (Figure 3), culture was performed to recover isolates that could be both genotyped and serotyped (39). Despite the use of specialized media required to support the growth of fastidious leptospires from cattle (17, 18), recovery of viable isolates was limited to just two sample collection days, and only preferentially when inoculated growth media were maintained at 37°C, contrary to traditional growth conditions of 28–30°C (14). However, once *in vitro* growth was established, strain MN900 was then readily maintained at both 37 and 29°C (data not shown). Notably, at all times dairy cow MN900 was seronegative for serovar Tarassovi in the MAT panel, including the reference strain and the isolate cultured from urine. The excretion of pathogenic leptospires from seronegative bovine hosts is not uncommon and highlights the limited utility of the MAT to detect asymptomatic carrier animals (2, 7, 8). Leptospires routinely colonize renal tubules as well as the genital tract; though not specifically examined here, it is also possible that excretion of leptospires is derived from the genital tract which in turn may have a limited role in generating an MAT detectable serology response (40).

The complete genome of strain MN900 not only confirmed that it belonged to *L*. *borgpetersenii* but that it was most closely related to multiple isolates from serovar Tarassovi (Figure 4, Supplementary Figure S1). Notably, the most closely related genome sequence was derived from a human serovar Tarassovi isolate cultured in 1942 in Australia (GCA_015163015.1), highlighting the widespread dissemination of this serovar. The serovar status of strain MN900 was confirmed with monoclonal antibodies. *L*. *borgpetersenii* serovar Tarassovi strain MN900 has a similar protein profile to that of other serovars within the same species (Figures 7A,B). In contrast, and as expected given that strain MN900 belongs to a different serogroup to that of serovar Hardjo strain TC129 and serogroup Ballum strain LR131, different LPS profiles were detected among these three strains.

Lipopolysaccharide of pathogenic leptospires is unusual since it shows a different profile to that of typical Gram-negative bacteria, is much less toxic, and elicits host species-specific Toll-like receptor (TLR) 2 and TLR 4 responses (41). LPS of *L*. *borgpetersenii* serovar Hardjo fails to elicit an inflammatory response in bovine endometrial epithelial cells (42). Nevertheless, leptospiral LPS is a major component of the outer membrane of pathogenic leptospires and acts as a protective antigen providing protection from homologous challenge but not from serovars in different serogroups (43). Since bovine bacterin vaccines only include serovars known to be associated with bovine leptospirosis, no U.S. leptospirosis vaccines contain serovar Tarassovi. In New Zealand, bovine leptospirosis is typically associated with serovars Hardjo and Pomona; however, recent studies have determined that serovar Tarassovi is not only an emerging serovar associated with disease in the dairy cattle population but is additionally an emerging public health risk (6, 44).

*L*. *borgpetersenii* serovar Tarassovi strain MN900 has an unusual and atypical morphology for a pathogenic leptospire when visualized by dark-field microscopy, whether cultured at 29 or 37°C. When compared to that of other strains of *L*. *borgpetersenii*, albeit in different serogroups, strain MN900 is much less motile, does not appear as tightly coiled, and lacks the classic hook shape at one or both ends (Supplementary Videos). Scanning electron microscopy confirmed that strain MN900 lacks any hooked ends but is tightly coiled (Figure 6D).

Leptospiruria in cows can be intermittent, and leptospires can be isolated from kidneys long after apparent leptospiruria has ceased (45). Multiple diagnostic approaches (FAT, PCR, and culture) were performed to detect subclinical urinary shedding of leptospires in dairy cow MN900 (46). Multiple diagnostic approaches are recommended since previous studies have demonstrated, in both experimentally and naturally infected cows, that none of these assays have a sensitivity of 100% and there is variation among samples and cattle, and at least two assays should be used to maximize the sensitivity of detection of leptospires in bovine urine (46, 47). Urinary shedding was sustained, though intermittent, for 18 weeks and as quantified by qPCR. Reasons for cessation of shedding after 18 weeks are not clear but may be due to the change in environment and/or removal of stress associated with milk production. Our analysis was limited to that of urine; carriage and disease transmission *via* colonization of the genital tract for serovar Tarassovi in cattle remains to be determined.

In conclusion, our results identified a fastidious species and serovar of *Leptospira* in a U.S. dairy cow that can be persistently (albeit intermittently) excreted *via* urine, which is not represented in any U.S. animal vaccine. Levels of the prevalence of *L. borgpetersenii* serovar Tarassovi in U.S. dairy cows remain to be determined, as does the identification of reservoir hosts of infection. The culture of *L*. *borgpetersenii* serovar Tarassovi in U.S. wildlife animals, including deer, has not yet been described. This work highlights the importance of culture and concomitant genotyping and serotyping to accurately classify leptospires, and as required to design efficacious vaccine and diagnostic strategies to not only limit animal disease but reduce zoonotic risk.

## Data Availability Statement

The datasets presented in this study can be found in online repositories. The names of the repository/repositories and accession number(s) can be found in the article/Supplementary Material.

## Ethics Statement

The animal study was reviewed and approved by the Animal Care and Use Committee at the National Animal Disease Center, Ames, Iowa.

## Author Contributions

JN: conceptualization. CH, KL, EP, DB, PC, MG, HL, NS, and JS: methodology. CH, KL, EP, DB, PC, MG, HL, NS, JS, LS, DW, and JN: formal analysis, writing—review and editing. MG, LS, DW, and JN: resources. CH, NS, JS, MG, EP, and JN: figures. CH and JN: writing—original draft preparation. All authors have read and agreed to the published version of the manuscript.

## Funding

This research was supported in part by an appointment to the Animal and Plant Health Inspection Service (APHIS) Research Participation Program administered by the Oak Ridge Institute for Science and Education (ORISE) through an interagency agreement between the U.S. Department of Energy (DOE) and the U.S. Department of Agriculture (USDA). ORISE is managed by ORAU under DOE contract number DE-SC0014664. USDA is an equal opportunity provider and employer. Mention of trade names or commercial products in this publication is solely for the purpose of providing specific information and does not imply recommendation or endorsement by the U.S. Department of Agriculture.

## Conflict of Interest

The authors declare that the research was conducted in the absence of any commercial or financial relationships that could be construed as a potential conflict of interest.

## Publisher's Note

All claims expressed in this article are solely those of the authors and do not necessarily represent those of their affiliated organizations, or those of the publisher, the editors and the reviewers. Any product that may be evaluated in this article, or claim that may be made by its manufacturer, is not guaranteed or endorsed by the publisher.

## Supplementary Material

Supplementary Figure 1 Phylogeny of *Leptospira borgpetersenii* strain MN900 based on complete whole genome sequence analysis.

Supplementary Videos Show representative motility of *L. borgpetersenii* serovar Tarassovi strain MN900, *L. borgpetersenii* serovar Hardjo strain TC129, and *L*. *borgpetersenii* serogroup Ballum strain LR131.

Supplementary Table 1 Microscopic agglutination test (MAT) panel of antigens. ND, Not determined, ^*^Obtained in this study.

Supplementary Table 2 Panel of reference antisera used to identify the serogroup of *L*. *borgpetersenii* strain MN900.

Supplementary Table 3 Complete results from samples collected from dairy cow MN900.
