# Supplementary figures and images for: Bovine Leptospirosis Due to Persistent Renal Carriage of Leptospira borgpetersenii Serovar Tarassovi
Source: Front Vet Sci. 2022 Apr 5;9:848664. doi: 10.3389/fvets.2022.848664 (PMC9019706; doi:10.3389/fvets.2022.848664)

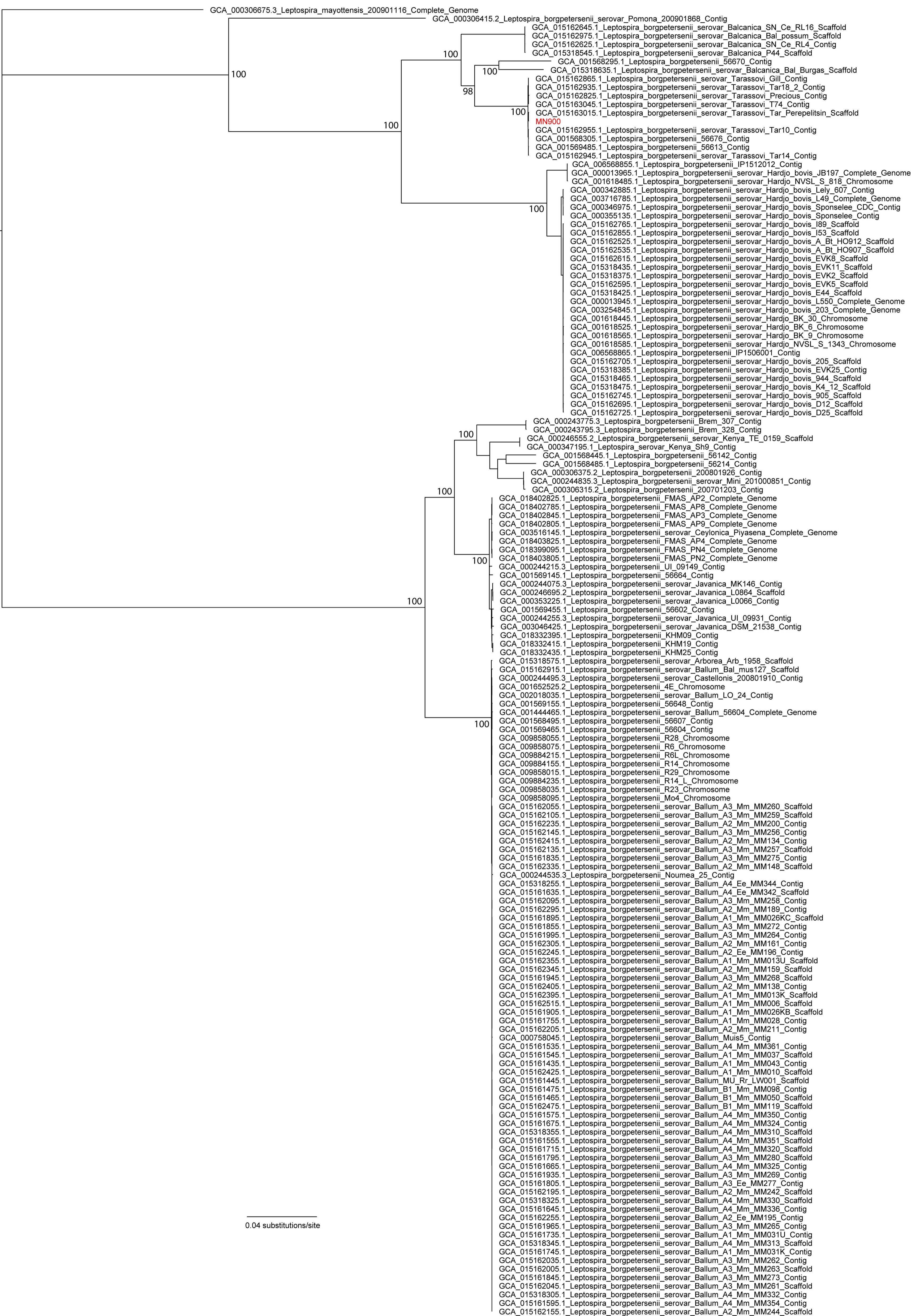

Supplement: Supplementary Figure 1 — Phylogeny of Leptospira borgpetersenii strain MN900 based on complete whole genome sequence analysis. [file Image_1.pdf]
