# Supplementary material for: Bovine Leptospirosis Due to Persistent Renal Carriage of Leptospira borgpetersenii Serovar Tarassovi
Source: Front Vet Sci. 2022 Apr 5;9:848664. doi: 10.3389/fvets.2022.848664 (PMC9019706; doi:10.3389/fvets.2022.848664)
Supplement: Supplementary Table 2 — Panel of reference antisera used to identify the serogroup of L. borgpetersenii strain MN900. [file Table_2.DOCX]

**SUPPLEMENTARY TABLE 2:** Panel of reference antisera used to identify the serogroup of *L*. *borgpetersenii* strain MN900.

| **Species** | **Serogroup** | **Serovar** | **Strain** |
| --- | --- | --- | --- |
| *L. interrogans* | Australis | Bratislava | Jez Bratislava |
| *L. interrogans* | Australis | Australis | Ballico |
| *L. interrogans* | Autumnalis | Autumnalis | Akiyami A |
| *L. borgpetersenii* | Ballum | Ballum | S 102 |
| *L. interrogans* | Bataviae | Bataviae | Van Tienen |
| *L. interrogans* | Canicola | Canicola | H. Utrecht IV |
| *L. interrogans* | Grippotyphosa | Grippotyphosa | Andaman |
| *L. interrogans* | Hebdomadis | Hebdomadis | Hebdomadis |
| *L. interrogans* | Icterohaemorrhagiae | Copenhageni | M 20 |
| *L. interrogans* | Mini | Szwajizak | Szwajizak |
| *L. interrogans* | Pomona | Pomona | Pomona |
| *L. interrogans* | Pyrogenes | Pyrogenes | Salinem |
| *L. interrogans* | Sejroe | Hardjo | Hardjoprajitno |
| *L. borgpetersenii* | Sejroe | Sejroe | M 84 |
| *L. borgpetersenii* | Tarassovi | Tarassovi | Perepelitsin |
